# Effect of pulmonary rehabilitation on heart rate recovery in adult individuals with asthma or chronic obstructive pulmonary disease

**DOI:** 10.3389/fphar.2022.956549

**Published:** 2022-09-27

**Authors:** Elisabetta Zampogna, Nicolino Ambrosino, Federico Mattia Oliva, Monica Rudi, Giovanni Sotgiu, Laura Saderi, Antonio Spanevello, Dina Visca

**Affiliations:** ^1^ Division of Pulmonary Rehabilitation, Istituti Clinici Scientifici Maugeri, IRCCS, Tradate, Italy; ^2^ Division of Pulmonary Rehabilitation, Istituti Clinici Scientifici Maugeri, IRCCS, Montescano, Italy; ^3^ Department of Medical, Surgical and Experimental Sciences, Clinical Epidemiology and Medical Statistics Unit, University of Sassari, Sassari, Italy; ^4^ Department of Medicine and Surgery, University of Insubria, Varese, Italy

**Keywords:** exercise training, exercise capacity, autonomic nervous system, heart rate variability, rehabilitation

## Abstract

**Introduction:** Heart rate recovery (HRR) after exercise is a marker of disease severity and prognosis in cardiovascular and respiratory disorders. More than 30% of adult individuals with asthma may show a slow HRR. Pulmonary rehabilitation improves exercise capacity in individuals with asthma or chronic obstructive pulmonary disease (COPD).

**Aim:** The study aimed to evaluate the effect of pulmonary rehabilitation on HRR in individuals with asthma as compared to those with COPD.

**Methods:** Retrospective analysis of HRR one minute after the six-minute walking test (6MWT) was performed before and after an exercise training program. The COPD Assessment Test (CAT), Barthel Index-Dyspnea (BI-D), Medical Research Council (MRC) score for dyspnea, and the Five-Times-Sit-to-Stand test (5STS) were also assessed as secondary outcome measures.

**Results:** Slow HRR prevalence was significantly lower in individuals with asthma than with COPD (29.1 vs. 46.7%, respectively: *p* = 0.003). Post-program HRR did not change in more than 70% of individuals in either population and improved in 16% of both populations, whereas it actually worsened in 12 and 10% of individuals with asthma and COPD, respectively. The outcome measures significantly improved in both populations, irrespective of baseline HRR.

**Conclusion:** In individuals with asthma or COPD, exercise training does not significantly improve HRR.

## Introduction

Autonomic nerve function impairment refers to sympathetic nerve over-activation and blunted parasympathetic nerve function. Heart rate recovery (HRR), defined as a reduction in the heart rate within the following minutes after the end of exercise, is a simple and reliable tool to assess autonomic nerve function (Arai et al., 1989; Pierpont and Voth, 2004). Slow HRR has been defined as a reduction in HR 1 minute after the end of exercise from HR at peak exercise (HR_peak_) of less than 12 or 14 bpm (Cole et al., 1999). There is evidence suggesting that slow HRR is a prognostic marker in different diseases, such as coronary artery disease, heart failure, and chronic thromboembolic pulmonary hypertension, as well as in individuals with chronic obstructive pulmonary disease (COPD) (Morshedi-Meibodi et al., 2002; Seshadri et al., 2004; Lacasse et al., 2005; Nanas et al., 2006; Lachman et al., 2018; Jin et al., 2022).

The HRR in individuals with chronic respiratory diseases has been assessed by means of maximal exercise tests or field tests such as the six-minute walk test (6MWT) (Rodríguez et al., 2017; Pereira et al., 2021; Zhao et al., 2021; Zampogna et al., 2022). The prevalence of slow HRR is estimated to be lower in individuals with asthma than in those with chronic obstructive pulmonary disease (COPD) (Zampogna et al., 2022). Individuals with COPD who exhibited slow HRR after the 6MWT exhibited worse exercise capacity and a more pronounced sedentary lifestyle and worse functional status than those with normal HRR (Morita et al., 2018).

Pulmonary rehabilitation including exercise training has strong evidence of effectiveness in improving dyspnea and fatigue, exercise capacity, and health-related quality of life [HRQL) in individuals with COPD and other chronic respiratory diseases including asthma (Zampogna et al., 2019; Paneroni et al., 2020). Therefore, current guidelines recommend pulmonary rehabilitation, including exercise training, in the comprehensive management of these individuals (Global Initiative for Chronic Obstructive Lung Disease, 2021). In individuals with COPD, HRR improves following exercise training programs or through oxygen supplementation in those with chronic hypoxemia (Scalvini et al., 1999; Gimeno-Santos et al., 2014). Obese children with bronchial asthma undergoing pulmonary rehabilitation showed a significant improvement in cardiorespiratory function and HRR, as well as in the inflammatory profile and functional capacity (Elnaggar et al., 2021). However, to the best of our knowledge, no study has evaluated the effects of exercise training on HRR in adult individuals with asthma. Therefore, the aim of this study was to evaluate the effect of an in-hospital pulmonary rehabilitation program including exercise training on HRR in a large population of adult individuals with asthma as compared to individuals with COPD.

## Material and methods

This retrospective study was approved by the Ethics Committee of Istituti Clinici Scientifici (ICS) Maugeri IRCCS (#2279). In this retrospective study, participants did not provide any specific written informed consent; however, at admission, they gave—in advance—informed consent for the scientific use of their clinical data. As a retrospective analysis, the study was not registered.

### Participants

The study was conducted on the Automated Integrated Health Care Record database of individuals with a reported diagnosis of COPD or asthma consecutively admitted between January 2019 and December 2021, to the Respiratory Unit of ICS Maugeri IRCCS of Tradate, Italy, a referral hospital for pulmonary rehabilitation, diagnosis, and care of chronic diseases (Maestri et al., 2019). Participants had been admitted when symptomatic despite optimized therapy, or when reported at least two exacerbations in the previous 12 months (an exacerbation within 30 days was classified as “recent”).

Inclusion criteria were as follows: age ≥18 years; confirmed diagnosis of asthma or COPD, according to the Global Initiative for Asthma (GINA) (Global Initiative for Asthma, 2021) or Global Initiative for Chronic Obstructive Lung Disease (GOLD) (Global Initiative for Chronic Obstructive Lung Disease, 2021) guidelines respectively; and availability of results of the 6MWT performed at admission and at discharge. Exclusion criteria were as follows: asthma-COPD overlap syndrome (Tu et al., 2021), diagnosis of obstructive sleep apnea, or orthopedic or neurological diseases preventing the performance of the exercise test or training at the target intensity.

### Measurements

The following data were recorded, and measurements were assessed at admission:

• Demographics, anthropometrics, diagnoses and severity of asthma or COPD according to guidelines (Global Initiative for Chronic Obstructive Lung Disease, 2021; Pereira et al., 2021), and comorbidities rated with the Cumulative Illness Rating Scale (CIRS) (Linn et al., 1968).
• Drug therapy (inhaled and cardiovascular drugs).
• Arterial blood gases were assessed on blood samples from the radial artery with an ABL 825 gas analyzer (Radiometer, Copenhagen, Denmark) in the sitting position.
• Dynamic lung volumes according to standards using the predicted values of Quanjer (Culver et al., 2017; Quanjer et al., 2012).
• Impact of disease by means of the COPD Assessment Test (CAT) (Kurashima et al., 2016). The CAT score ranges from 0 to 40 (higher scores indicate a more severe impact of disease on life). A reduction by 2 points is considered the minimal clinically important difference (MCID) in individuals with COPD (Kon et al., 2014).
• Dyspnea was assessed by the Barthel Index-Dyspnea (BI-D) (Vitacca et al., 2016). Total scores range from 0 (absence of dyspnea) to 100 (most severe dyspnea). A 9-point reduction is considered the MCID in patients with COPD without chronic respiratory failure (Vitacca et al., 2020).
• Dyspnea was also assessed by means of the Medical Research Council (MRC) score (Fletcher et al., 1959). A one-point decrease is considered a value equivalent to the MCID for MRC (De Torres et al., 2002).
• Lower limb function was assessed by the Five-Times-Sit-to-Stand (5STS) test, and the estimated MCID is 1.7s (Jones et al., 2013; Zampogna et al., 2021a).
• The 6MWT, according to standards using the reference values by Enright et al. (Holland et al., 2014; Enright and Sherrill, 1998), was used under pulse oximetry (SpO_2_) monitoring (Nonin PalmSAT^®^ 2500). The following parameters were recorded: ΔHR: HR_peak_—HR baseline; SpO_2 nadir_; and exercise-induced oxygen desaturation (EID): SpO_2 baseline_ - SpO_2 nadir_ ≥ 4% (Poulain et al., 2003). HRR was assessed as HR_peak_ - HR at first minute after the end of the test. Slow HRR was defined as < 12 bpm. The maximal predicted HR was calculated as 220-age. The MCID of 6MWT has been defined as a 30-m post treatment change in individuals with COPD (Holland et al., 2014) and 26 m in individuals with asthma (Zampogna et al., 2021b).

### Pulmonary rehabilitation program

Both populations received the same standard pulmonary rehabilitation program offered by a team of chest physicians, nurses, physical therapists, dietitians, and psychologists, as previously described (Zampogna et al., 2019; Maestri et al., 2019). The 3-week in-patient program included exercise training, peripheral muscle mobilization, educational sessions on medical issues and correct inhalation therapy, nutritional and psychological counseling, and individualized diet when appropriate. Nurses supervised the respect of drug prescriptions and correct use of inhaled therapy.

Endurance training consisted of at least twelve 30-min daily sessions of incremental exercise training supervised by a physiotherapist according to (Maltais et al., 1997) and continuous cycling at 50–70% of maximal load calculated on baseline 6MWT according to the Hill’s formula (Hill et al., 2008). Workload adjustments were based on perceived dyspnea and muscle fatigue, as measured on the modified Borg scale (Borg, 1982). A score <3 resulted in a 5 W increase, and a score >5, in a 5 W reduction, while a score of 4 or 5 of the workload remained unchanged. Peripheral muscle mobilization consisted of twelve 30-min daily sessions using free weights. The workload was increased by 0.5/1 kg when subjects scored their muscular fatigue <3 on a modified 10-point Borg Scale. The workload was unchanged if the Borg score was 4 or 5 and was reduced by 0.5 kg for scores >5.

### Statistical analysis

An *ad hoc* excel form aimed at collecting epidemiological, demographic, and clinical variables was prepared. A descriptive analysis of the variables collected for individuals with asthma and COPD was performed. Qualitative variables were described with absolute and relative (percentage) frequencies, whereas quantitative variables were summarized with means and standard deviations (SD) or medians and interquartile ranges (IQR) in the case of parametric and non-parametric distributions, respectively. Differences in qualitative variables were analyzed with chi-squared or Fisher’s exact tests when appropriate. Quantitative variables with normal distribution were compared using Student’s t-test, whereas those with non-normal distribution, using Mann-Whitney test. A two-tailed *p*-value <0.05 was considered statistically significant. All statistical analyses were performed by STATA statistical software version 17 (StataCorp LCC, Texas, United States).

## Results

**FIGURE 1:**
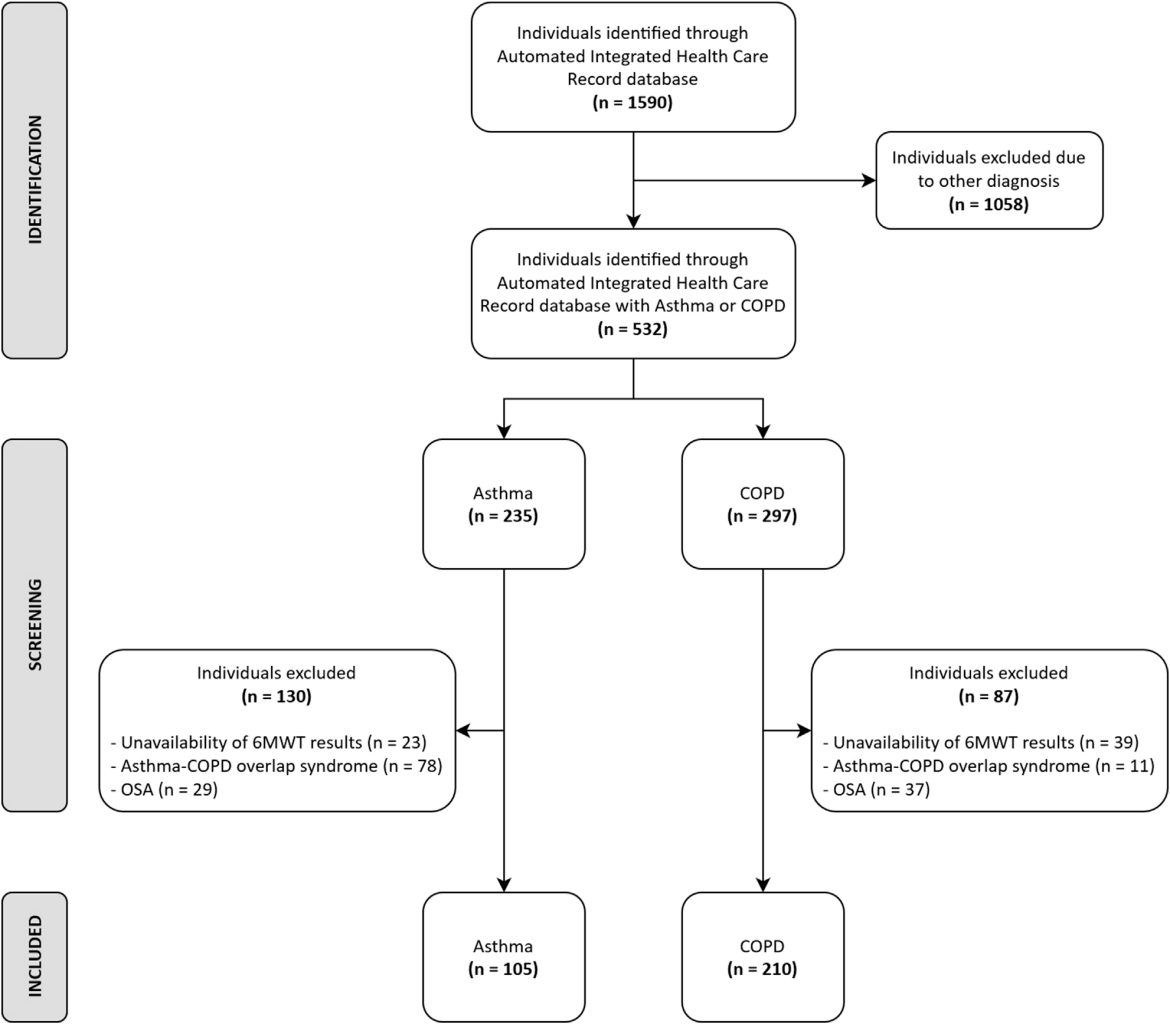
Patient inclusion flowchart.

Out of 1,590 individuals admitted in the study period, data from 315 (105 suffering from asthma and 210 from COPD) participants fulfilled the inclusion criteria (Figure 1). The demographic, anthropometric, physiological, and clinical characteristics of participants are shown in Table 1. A recent exacerbation (within 30 days) was reported in 14 and 26% of participants with asthma or COPD, respectively. As expected, individuals with asthma showed a higher prevalence of females, were significantly younger, showed a higher body mass index (BMI), suffered from less severe chronic airway obstruction, reported less dyspnea, walked significantly longer, and showed a lower prevalence of EID during the 6MWT than individuals with COPD, without any significant differences in the CAT and 5STS.

**TABLE 1:** Demographic, anthropometric, physiological, and clinical characteristics of participants.

Variable		Total (*n* = 315)	Asthma (*n* = 105)	COPD (*n* = 210)	*p*-value
Males, *n* (%)		168 (53.3)	33 (31.4)	135 (64.3)	<0.0001
Age, years		70 (62–77)	66 (55–74)	71 (65–78)	0.0001
BMI, kg/m^2^		26.4 (22.9–29.8)	27.6 (24.1–31.0)	25.6 (22.4–29.2)	0.004
Class BMI, *n* (%)	≤24.9 kg/m^2^	126 (40.0)	32 (30.5)	94 (44.8)	0.02
	25.0–29 kg/m^2^	114 (36.2)	40 (38.1)	74 (35.2)	
	≥30.0 kg/m^2^	75 (23.8)	33 (31.4)	42 (20.0)	
CIRS comorbidity, score		1.5 (1.4–1.7)	1.5 (1.4–1.7)	1.5 (1.5–1.7)	0.99
CIRS severity, score		3.2 ± 1.4	3.2 ± 1.4	3.2 ± 1.4	0.29
Cardiovascular diseases, *n* (%)		74 (23.5)	14 (13.3)	60 (28.6)	0.003
Diabetes mellitus, *n* (%)		247 (7.6)	7 (6.7)	17 (8.1)	0.65
Hypertension, *n* (%)		119 (37.8)	41 (39.1)	78 (37.1)	0.74
Inhaled therapy, *n* (%)		307 (97.4)	104 (99.1)	203 (96.7)	0.28
ICS		1 (0.3)	1 (1.0)	0 (0.0)	0.33
ICS plus LABA		242 (76.8)	104 (99.1)	138 (65.7)	<0.0001
LABA plus LAMA		65 (20.6)	0 (0.0)	65 (31.0)	<0.0001
Oral steroid plus LABA		27 (8.69	27 (25.7))	0 (0.0)	<0.0001
Cardiovascular therapy, *n* (%)		181 (57.5)	54 (51.4)	127 (60.5)	0.13
Beta blockers		83 (26.4)	18 (17.1)	65 (31.0)	0.009
Calcium channel blockers		67 (21.3)	20 (19.1)	47 (22.4)	0.50
Renin–angiotensin–aldosterone system inhibitors		135 (42.9)	43 (41.0)	92 (43.8)	0.63
GINA, *n* (%)	1		1 (1.0)	-	-
	2		3 (2.9)	-	
	3		12 (11.4)	-	
	4		25 (23.8)	-	
	5		64 (61.0)	-	
GOLD, *n* (%)	1		-	34 (16.1)	-
	2		-	77 (36.6)	
	3		-	69 (32.8)	
	4		-	29 (13.8)	
Oxygen therapy, *n* (%)	No	201 (63.8)	93 (88.6)	109 (51.9)	<0.0001
	Yes	114 (36.2)	12 (11.4)	101 (48.1)	
PaO_2_, mm Hg		73.3 (67.1–82.0)	80.6 (72.2–88.9)	71.2 (65.2–77.9)	<0.0001
PaCO_2_, mm Hg		36.9 (34.4–40.6)	36.4 (34.4–38.4)	37.4 (34.5–41.8)	0.005
SaO_2_%		95.1 ± 1.9	95.7 ± 1.7	94.8 ± 2.0	0.0001
FEV1, liters		1.5 (1.0–2.1)	1.9 (1.4–2.3)	1.2 (0.9–1.7)	<0.0001
FEV1, % predicted		60 (42–80)	77 (59–93)	50 (38–68)	<0.0001
FVC, liters		2.7 (2.0–3.3)	2.8 (2.2–3.4)	2.6 (2.0–3.3)	0.17
FVC, % predicted		84.1 ± 20.8	89.2 ± 18.6	81.4 ± 21.4	0.002
FEV1/FVC%		57.0 (44.1–66.7)	67.8 (58.5–75.9)	51.9 (39.6–61.5)	<0.0001
Outcome measures					
MRC, score, *n* (%)	0	15 (6.0)	4 (5.1)	11 (6.4)	0.69
	1	69 (27.6)	32 (40.5)	37 (21.6)	0.002
	2	68 (27.2)	20 (25.3)	48 (28.1)	0.64
	3	52 (20.8)	15 (19.0)	37 (21.6)	0.64
	4	46 (18.4)	8 (10.1)	38 (22.2)	0.02
Missing data, *n* (%)		65 (20.6)	26 (24.8)	39 (18.8)	-
Bi-D, score		14.5 (8–22)	12 (7–20)	16 (9–23	0.001
Missing data		11 (3.5)	4 (3.8)	7 (3.3)	-
CAT, score		15 (9–21)	15 (9–21)	14 (9–21)	0.92
Missing data		68 (21.6)	20 (19.0)	48 (22.9)	-
5STS, sec		14.7 (12.6–19.0	14.7 (12.8–18.9)	14.9 (12.6–19.4) -104	0.84
Missing data		160 (50.8)	56 (53.3)	104 (49.5)	-
6MWT, m		412 (324–483)	463 (375–514)	384 (300–461)	<0.0001
6MWT, % predicted		85.1 (70.5–99.3)	93.6 (80.0–106.6)	80.3 (66.0–95.5)	<0.0001
HR_peak_, bpm		107.6 ± 14.9	111.3 ± 13.7	105.7 ± 15.2	0.002
ΔHR, bpm		30 (23–40)	34 (27–44)	29 (22–37)	0.0001
SpO_2 mean_%		92 (90–95)	95 (93–96)	91 (89–93)	<0.0001
SpO_2 nadir_%		90 (86–92)	92 (91–94)	88 (84–91)	<0.0001
EDI, *n* %		198 (62.9)	42 (40)	156 (74.3)	<0.0001
HRR, *n* (%)	>12 bpm	185 (59.1)	73 (70.9)	112 (53.3)	0.003
	≤12 bpm	128 (40.9)	30 (29.1)	98 (46.7)	

Data as numbers (*n*) and percentage (%), median [IQR], or mean ± SD. Missing data specifically indicated. Abbreviations: COPD, chronic obstructive pulmonary disease; BMI, body mass index; CIRS, Charlson Comorbity Index; ICS, inhaled steroid; LABA, long-acting beta agonist; LAMA, long-acting muscarinic antagonist; GINA, Global Initiative for Asthma; GOLD, Global Initiative for Chronic Obstructive Lung Disease; PaO_2_, arterial oxygen tension; PaCO_2_, arterial carbon dioxide tension; SaO_2_, arterial oxygen saturation; FEV1, forced expiratory volume at 1 s; FVC, forced vital capacity; MRC, Medical Research Council; BId, Barthel Index-Dyspnea; CAT, COPD Assessment Test; 5STS, Five-Times- Sit-to-Stand test; 6MWT, six-minute walk test; HR, heart rate; ΔHR, peak–baseline HR; SpO_2_, peripheral oxygen saturation; EDI, exercise-induced desaturation; HRR, heart rate recovery.

As also shown in Table 1 and as expected, almost all individuals with asthma and COPD were using inhaled long-acting beta agonist (LABA). Individuals with COPD used significantly more long-acting muscarinic agents (LAMA), whereas those with asthma used more inhaled steroids (ICS). Individuals with COPD showed a higher prevalence of the use of beta-blockers, whereas there was no significant difference between groups in the use of calcium channel blockers or renin–angiotensin–aldosterone system inhibitors.

The prevalence of slow HRR was significantly lower in individuals with asthma than with COPD (29.1 vs. 46.7% respectively, *p* = 0.003).

Table 2 shows the demographic, anthropometric, physiological, and clinical characteristics of participants according to HRR. In participants with asthma, the only significant differences between people with slow and normal HRR were in HR_peak_ and ΔHR during the 6MWT. Individuals with COPD and slow HRR suffered from significantly more severe airway obstruction, symptoms, and reduced exercise tolerance than those with normal HRR. There was no significant difference in the prevalence of slow HRR between individuals with and without recent exacerbations in either population (*p* = 0.29 and 0.10 in individuals with asthma or COPD, respectively).

**TABLE 2:** Baseline characteristics according to HRR.

		Asthma (*n* = 105)			COPD (*n* = 210)		
		HRR1 > 12	HRR1 ≤ 12	*p*-value	HRR1 > 12	HRR1 ≤ 12	*p*-value
*n* (%)		73 (70.9)	30 (29.1)	**-**	112 (53.3)	98 (46.7)	
Males, *n* (%)		24 (32.9)	9 (30.0)	0.78	69 (61.6)	66 (67.4)	0.39
Age, years		64 (54–73)	71.5 (59–79)	0.07	71 (65–77)	71 (64–78)	0.94
BMI, kg/m^2^		27.6 (24.3–31.4)	28.7 (24.3–31.0)	0.89	25.7 (4.8)	26.2 ()5.6	0.46
Class BMI, *n* (%)	≤24.9 kg/m^2^	21 (28.8)	9 (30.0)	0.96	51 (45.5)	43 (43.9)	0.14
	25.0–29.Kg/m^2^	29 (39.7)	11 (36.7)		44 (39.3)	30 (30.6)	
	≥30.0 kg/m^2^	23 (31.5)	10 (33.3)		17 (15.2)	25 (25.5)	
CIRS comorbidity, score		1.5 ± 0.2	1.6 ± 0.3	0.71	1.5 (1.4–1.7)	1.6 (1.5–1.7)	0.88
CIRS severity, score		3 (2–4)	3 (2–4)	0.88	3.2 ± 1.5	3.2 ± 1.4	0.96
Cardiovascular disease, *n* (%)		8 (11.0)	5 (16.7)	0.43	33 (29.5)	27 (27.6)	0.76
Diabetes mellitus, *n* (%)		7 (9.6)	0 (0.0)	0.10	8 (7.1)	9 (9.2)	0.59
Hypertension, *n* (%)		28 (38.4)	12 (40.0)	0.88	40 (35.7)	38 (38.8)	0.65
Step GINA, *n* (%)	1	1 (1.4)	0 (0.0)	0.26	-	-	-
	2	2 (2.7)	0 (0.0)		-	-	
	3	6 (8.2)	6 (20.0)		-	-	
	4	16 (21.9)	9 (30.0)		-	-	
	5	48 (65.8)	15 (50.0)		-	-	
GOLD, *n* (%)	1	-	-	-	16 (15.1)	12 (13.5)	**0.03**
	2	-	-		49 (46.2)	24 (27.0)	
	3	-	-		30 (28.3)	37 (41.6)	
	4	-	-		11 (10.4)	16 (18.0)	
Oxygen therapy, *n* (%)	No	64 (87.7)	26 (86.7)	1.00	64 (57.1)	45 (45.9)	0.26
	24 h	4 (5.5)	2 (6.7)		21 (18.8)	23 (23.5)	
	15/18 h	2 (2.7)	1 (3.3)		13 (11.6)	12 (12.2)	
	Night	3 (4.1)	1 (3.3)		14 (12.5)	15 (15.3)	
PaO_2_, mm Hg		82.1 ± 11.7	79.6 ± 10.7	0.32	71.9 (67.0–79.3)	68.2 (63.4–75.9)	**0.01**
PaCO_2_, mm Hg		36.1 (33.7–37.9)	37.0 (34.6–39.2)	0.24	37.1 (34.4–40.7)	38.3 (34.9–44.1)	0.08
SaO_2_%		95.8 ± 1.8	95.7 ± 1.4	0.78	94.6 ± 1.9	94.6 ± 2.2	0.84
FEV1, liters		1.9 (1.5–2.3)	1.8 (1.2–2.3)	0.32	1.3 (1.0–1.8)	1.2 (0.8–1.6)	**0.05**
FEV1, % predicted		76.3 ± 22.0	76.4 ± 25.8	0.98	57 (41–69)	46 (35–66)	**0.03**
FVC, liters		2.9 ± 0.8	2.7 ± 1.0	0.24	2.6 (2.0–3.4)	2.5 (2.3–3.2)	0.51
FVC, % predicted		89.7 ± 17.6	89.8 ± 20.4	0.98	83 (71–99)	78 (64–88)	0.11
FEV1/FVC%		68.7 (60.2–76.4)	67.8 (54.7–72.7)	0.44	55.4 (42.8–62.3)	47.8 (36.2–60.7)	**0.03**
MRC, score, *n* (%)	0	3 (4.1)	1 (3.3)	0.65	6 (5.4)	5 (5.1)	**0.03**
	1	23 (31.5)	9 (30.0)		25 (22.3)	12 (12.2)	
	2	11 (15.1)	9 (30.0)		26 (23.2)	22 (22.5)	
	3	11 (15.1)	3 (10.0)		17 (15.2)	20 20.4)	
	4	7 (9.5)	1 (3.3)		15 (13.4)	23 (23.5)	
	Missing	18 (24.7)	7 (23.4)	-	23 (20.5)	16 (16.3)	-
BId, score		11 (7–20)	14 (5–20)	0.85	15 (9–22)	17.5 (10.5–29.0)	0.05
CAT, score		15.8 ± 8.4	14 ± 7.7	0.35	13 (8–19)	16 (10–22)	0.05
5STS, sec		14.7 (12.8–18.9)	15.8 (14.2–16.3)	0.92	14.4 (12.2–17.9)	15.6 (13.1–19.9)	0.13
6MWT, m		463 (386–525)	477 (325–514)	0.52	401 (335–475)	352 (290–430)	**0.002**
6MWT, % predicted		93 (78.7–109.0)	94.4 (80–103)	1.00	85.5 (70.5–99.3)	77.2 (58.9–90.3)	**0.008**
HR_peak_, bpm		113.4 ± 13.1	106.5 ± 14.7	**0.02**	108.3 ± 14.8	102.7 ± 15.1	**0.008**
ΔHR, bpm		37.1 ± 11.0	30.3 ± 10.7	**0.005**	30 (25–39)	25.5 (20–35)	**0.003**
SpO_2 mean_%		94.0 ± 2.8	93.8 ± 2.5	0.75	90.6 ± 3.0	90.9 ± 3.1	0.53
SpO_2 nadir_%		92 (91–94)	92 (90–95)	0.60	88.5 (86–91)	89 (85–91)	0.87
EID, *n* %		29 (39.7)	11 (36.7)	0.77	83 (74.1)	73 (74.5)	0.95

Data as numbers (*n*) and percentage (%), median [IQR], or mean ± SD. Abbreviations: COPD, chronic obstructive pulmonary disease; HRR, heart rate recovery; BMI, body mass index; CIRS, Charlson Comorbity Index; GINA, Global Initiative for Asthma; GOLD, Global Initiative for Chronic Obstructive Lung Disease; PaO_2_, arterial oxygen tension; PaCO_2_, arterial carbon dioxide tension; SaO_2_, arterial oxygen saturation; FEV1, forced expiratory volume at 1 s; FVC, forced vital capacity; MRC, Medical Research Council; BI-D, Barthel Index-Dyspnea; CAT, COPD assessment test; 5STS, Five-Times-Sit-to-Stand test; 6MWT, six-minute walk test; HR, heart rate; ΔHR, peak—baseline HR; SpO_2_, peripheral oxygen saturation; EID, exercise-induced oxygen desaturation.

*p*-values in bold are less than 0.05 and hence considered statistically significant.

**FIGURE 2:**
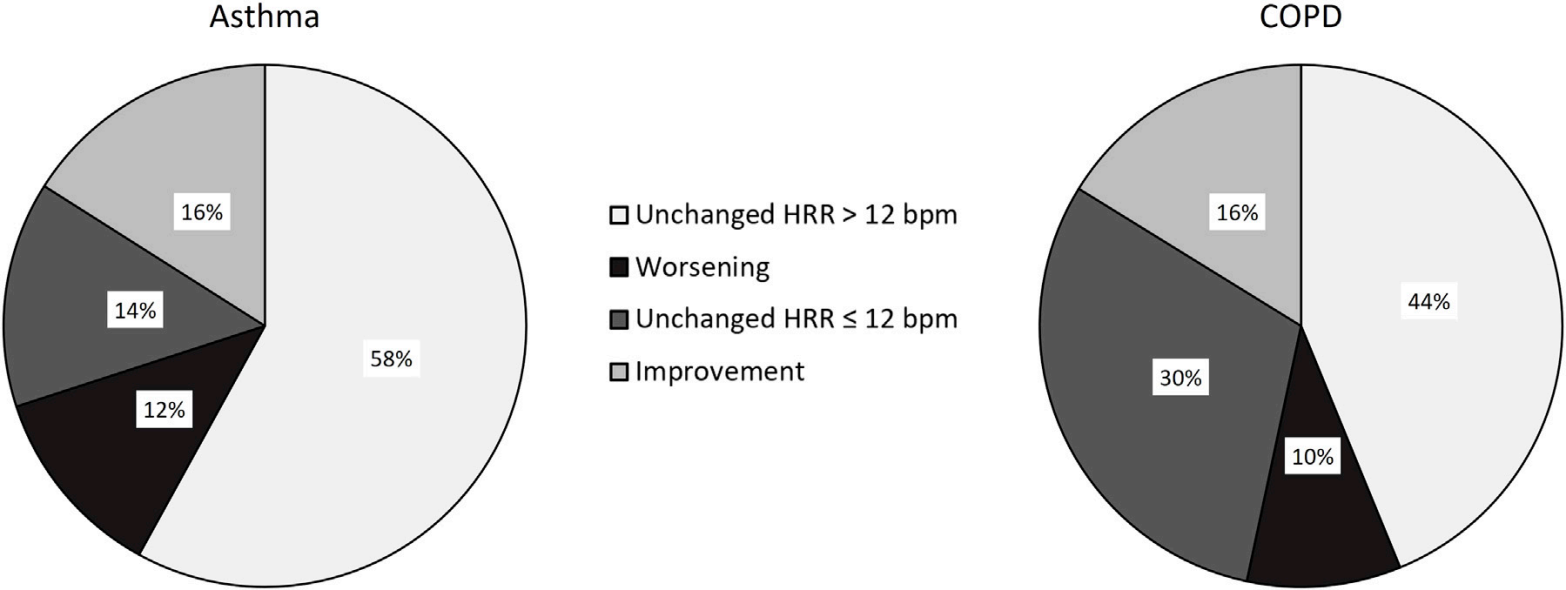
Changes in prevalence of HRR post-pulmonary rehabilitation in subjects with asthma and COPD.

Table 3 shows the post-rehabilitation changes in outcome measures and in prevalence of slow HRR in the populations in the study. As a whole, there was no significant change in the prevalence of slow HRR in either group. In detail, more than 70% of participants in either group showed no change in HRR, which improved (from slow to normal) only in 16% of participants in both populations, whereas in 12 and 10% of individuals with asthma and COPD, respectively, HRR actually worsened from normal to slow (Figure 2). There was no significant difference in the frequency of post-program slow HRR in individuals with and without recent exacerbation (*p* = 0.27 and 0.28 in individuals with asthma or COPD, respectively).

**TABLE 3:** Post-pulmonary rehabilitation values of and changes in outcome measures.

Variable		Asthma (*n* = 105)	*p*-value*	COPD (*n* = 210)	*p*-value§	*p*-value #
MRC, score, *n* (%)	0	30 (28.6)	**<0.0001**	42 (20.0)	**<0.0001**	**0.006**
	1	30 (28.6)		62 (29.5)		
	2	8 (7.6)		35 (16.7)		
	3	2 (1.9)		16 (7.6)		
	4	5 (4.7)		9 (4.3)		
	Missing	30 (28.6)	-	46 (21.9)	-	-
Δ MRC, score		−1 (−1; 0)	-	−1 (−2; 0)	-	0.79
BId, score		6 (2–11)	**<0.0001**	9 (6–16)	**<0.0001**	**0.004**
Δ BId, score		−5.5 (−13; -1)	-	−8 (−13; 0)	-	0.57
CAT, score		5 (3–10)	**<0.0001**	6 (3–12)	**<0.0001**	0.30
Δ CAT, score		−7 (−12; -4)	-	−6 (−10; -3)	-	0.53
5STS, sec		12.2 (10.9–14.3)	**<0.0001**	12.6 (10.5–14.9)	**<0.0001**	0.96
Δ 5STS, sec		−2.6 (−4.6; -0.2)	-	−3.0 (−4.9;-1.1)	-	0.25
6MWT, m		500 (435–558)	**<0.0001**	420 (353–499)	**<0.0001**	**<0.0001**
6MWT, % predicted		101.1 (89.6–111.4)	**<0.0001**	89.9 (75.7–103.7)	**<0.0001**	**<0.0001**
Δ 6MWT, meters		30 (10–65)	-	34 (1–65)	-	0.83
HR_peak_, bpm		117.4 ± 15.7	**<0.0001**	109.2 ± 16.3	**<0.0001**	**<0.0001**
ΔHR, bpm		40 (30–51)	**<0.0001**	31 (24–40)	**0.0005**	**<0.0001**
SaO_2 mean_%		95 (93–96)	**0.006**	91 (89–93)	0.08	**<0.0001**
SaO_2 nadir_%		93 (91–95)	**0.03**	89 (86–91)	**0.03**	**<0.0001**
EID, *n* %		35 (33.3)	0.31	158 (75.2)	0.83	**<0.0001**
HRR, *n* (%)	>12 bpm, *n* (%)	75 (74.3)	0.57	126 (60.0)	0.08	**0.01**
	≤12 bpm, *n* (%)	26 (25.7)	0.57	84 (40.0)	0.08	

Data as numbers (n) and percentage (%), median [IQR], or mean ± SD; * within the asthma group, the *p*-value of pre-post rehabilitation changes, # within the COPD group *p*-value of pre-post rehabilitation changes, # between groups *p*-value post-PR changes. Abbreviations: COPD, chronic obstructive pulmonary disease; MRC, Medical Research Council; ΔMRC, post-pre; BI-D, Barthel Index-dyspnea; ΔBI-d, post-pre; CAT, COPD assessment test; ΔCAT, post-pre; 5STS, Five-Times Sit-to-Stand test; Δ 5STS, pre-post; 6MWT, 6-min walking test; Δ6MWT, post-pre; HR, heart rate; ΔHR peak—baseline HR; SpO_2_, peripheral oxygen saturation; EID, exercise-induced desaturation; HRR, heart rate recovery.

*p*-values in bold are less than 0.05 and hence considered statistically significant.

There was no significant difference in the baseline prevalence of slow HRR, according to the use of beta-blockers or renin–angiotensin–aldosterone system inhibitors in either group (*p*-values ranging from 0.25 to 0.98). Individuals with COPD but not those with asthma under calcium channel blockers showed a higher baseline prevalence of slow HRR than those not using this therapy (59.6 vs. 42.9%, *p* = 0.04). There was no significant difference in post-training “improvers/worseners” rate according to any cardiovascular drug, or any comorbidity, including cardiovascular in either group.

As shown in Table 4, there was no significant difference in physiological effects of the program between participants with slow or normal HRR in the asthma population, whereas individuals with COPD and slow HRR improved slightly but significantly less the walked distance than those with normal HRR.

**TABLE 4:** Post pulmonary rehabilitation values of and changes in outcome measures according to HRR.

		Asthma (*n* = 105)			COPD (*n* = 210)		
		HRR >12	HRR ≤12	*p*-value	HRR> 12	HRR ≤12	*p*-value
MRC, score, *n* (%)	0	21 (28.8)	9 (30)	0.48	27 (24.1)	15 (15.30)	0.07
	1	19 (26.1)	11 (36.7)		32 (28.6)	30 (30.6)	
	2	5 (6.8)	2 (6.6)		17 (15.2)	18 (18.4)	
	3	2 (2.7)	0 (0.0)		7 (6.2)	9 (9.2)	
	4	5 (6.8)	0 (0.0)		3 (2.7)	6 (6.1)	
	Missing	21 (28.8)	8 (26.7)	-	26 (23.2)	20 (20.4)	-
Δ MRC, score		−1 (−1; 0)	−1 (−1; -1)	0.72	−1 (−1; 0)	−1 (−2; 0)	0.71
BId, score		5.5 (2–12)	6.5 (2.5–9.5)	0.98	8 (6–15)	12 (6–19)	0.30
Δ BId, score		−6 (-15; -2)	−5.5 (−10.5; -2)	0.55	−8 (-10; 0)	-8 (−15; 0)	0.48
CAT, score		5 (3–10)	5 (3–8)	0.67	5 (3–10)	8 (4–15)	**0.004**
Δ CAT, score		−7 (−13.5; −4)	−7 (−11; -3)	0.44	−7 (−10; −3)	−6 (−10; −3)	0.94
5STS, sec		12.1 (10.9–13.5)	12.9 (12.4–15.4)	0.21	11.7 (10.2–14.3)	12.9 (10.8–15.2)	**0.12**
Δ 5STS, sec		−3.2 (−5.6; −0.3)	−1.2 (−2.9;-0.7)	0.006	−2.9 (-4.7;-1.2)	−3.4 (−5.2;−1.0)	**0.73**
6MWT, m		500 (440–558)	490 (350–560)	0.53	445 (380–515)	385 (326–450)	**0.0004**
6MWT, % predicted		101.1 (87.0–110.3)	102.4 (90.0–111.7)	0.87	94.0 (81.0–107.3)	85.7 (72.5–95.5)	**0.002**
Δ 6MWT, meters		25 (5–63)	32 (15–65)	0.40	31 (2–59.5)	34 (0–67)	0.99
HR_peak_, bpm		117.9 ± 14.4	115.4 ± 18.0	0.46	111.1 ± 17.0	107.0 ± 15.2	**0.07**
ΔHR, bpm		42.0 ± 13.2	37.0 ± 15.4	0.10	33.5 (25–42)	28 (23–39)	0.03
SaO_2 mean_, %		95 (93–96)	95 (93–96)	0.63	90.6 ± 3.0	90.9 ± 3.1	0.53
SpO_2 nadir_, %		93 (91–95)	93 (91–95)	0.92	88.5 (86–91)	89 (85–91)	0.87
EID*, *n* %		24 (32.9)	10 (33.3)	0.96	85 (75.9)	73 (74.5)	0.81

Data as numbers (*n*) and percentage (%), median [IQR], or mean ± SD. Abbreviations: COPD, chronic obstructive pulmonary disease; HRR, heart rate recovery; MRC, Medical Research Council; ΔMRC, post-pre; BI-D, Barthel Index-Dyspnea; ΔBId, post-pre; CAT, COPD assessment test; ΔCAT, post-pre; 5STS, Five-Times Sit-to-Stand test; Δ 5STS, pre-post; 6MWT, six-minute walk test; Δ6MWT, post-pre; HR, heart rate; ΔHR, peak—baseline HR; SpO_2_, peripheral oxygen saturation; EID, exercise-induced desaturation.

*p*-values in bold are less than 0.05 and hence considered statistically significant.

As shown in Table 5 after the program, the MCID of each outcome measure was reached in different proportions, irrespective of HRR, either slow or normal, without any within or between group significant differences. There were no significant differences in pre- and post-training frequency of slow HRR between individuals with asthma or COPD reaching the MCID of 6MWT and those who did not (*p* values ranging from 0.710 to 0.926).

**TABLE 5:** Proportion of patients reaching the MCID after pulmonary rehabilitation.

	Asthma	COPD	*p*-value	Asthma	COPD	*p*-value
	HRR1 > 12			HRR1 ≤ 12		
MRC n (%)	34/52 (65.4)	59/87 (67.8)	0.77	17/22 (77.3)	52/78 (66.7)	0.34
BId n (%)	13/34 (38.2)	14/31 (45.2)	0.57	3/8 (37.5)	22/42 (52.4)	0.70
CAT n (%)	46/52 (88.5)	72/79 (91.1)	0.62	21/24 (87.5)	64/74 (86.5)	1.00
5STS n (%)	13/37 (35.1)	17/48 (35.4)	0.97	5/9 (55.6)	17/53 (32.1)	0.26
6MWT n (%)	36/73 (49.3)	61/112 (54.5)	0.49	16/30 (53.3)	54/98 (55.1)	0.87

Abbreviations: COPD, chronic obstructive pulmonary disease; HRR, heart rate recovery; MRC, Medical Research Council; MCID, minimum clinical important difference; BI-D, Barthel Index-dyspnea; CAT, COPD assessment test; 5STS, Five-Times Sit-to-Stand test; 6MWT, six-minute walk test.

## Discussion

To the best of our knowledge, while confirming our previous finding that individuals with adult asthma show a lower prevalence of slow HRR than individuals with COPD (Zampogna et al., 2022), this is the first study to evaluate the effects of pulmonary rehabilitation on HRR in adult individuals, with asthma. In these individuals, a pulmonary rehabilitation program, including exercise training, resulted in benefits in symptoms and exercise capacity but did not significantly improve the prevalence of slow HRR. There were no significant differences in outcome measures between individuals with pre-program slow or normal HRR. No significant difference was observed in the post-program size of HRR change between individuals with asthma or COPD.

In our study, despite the expected benefits of pulmonary rehabilitation on symptoms and exercise capacity, there were no significant improvements in the prevalence of slow HRR in either population. How can we explain our “negative results?” Cardiac autonomic dysfunction is present in individuals with mild to very severe COPD and is inversely related to the practice of physical activity (Morita et al., 2018; Delgado-Ortiz et al., 2022) The effect of the exercise training in HRR has been studied in individuals with moderate-to-severe COPD. After 8 weeks of interval training, HRR improved significantly, pre-training HRR being the only variable related to post-training HRR (Gimeno-Santos et al., 2014). In that study (Gimeno-Santos et al., 2014), the proportion of individuals with very severe airflow obstruction was higher than in our study (38 vs. 13%), with a higher baseline prevalence of slow HRR (63 vs. 47%). Other studies have shown that slow HRR is more prevalent in individuals with very severe COPD (Lacasse et al., 2005; Seshadri et al., 2004; Zhao et al., 2021). Therefore, we cannot exclude that the lack of substantial improvement in post-training HRR observed in our study might be due to less severe airflow limitation in our participants and the lower pre-training prevalence of slow HRR.

In addition, despite the mean values of post training changes in 6MWT indicating an overall training effect of the program, in both populations (Table 3), there was a relatively low prevalence of responders (individuals reaching the MCID of 6MWT, Table 5). Therefore, we might argue that the substantial lack of post-training improvement in the frequency of slow HRR might be due (also) to a lack of a training effect. However, there were no significant differences in pre- and post-training frequency of slow HRR between individuals of either group reaching the MCID of 6MWT and those who did not.

The lack of substantial post-training improvement in the frequency of slow HRR in adult individuals with asthma in our study cannot be explained by the cardiovascular drugs used. Indeed, there was no significant difference in the prevalence of post-training HRR “improvers/worseners” rate according to the use of cardiovascular drugs in either group.

Almost all individuals with asthma and COPD were using inhaled LABA. Whether this use has blunted any potential difference in the effect of training on HRR cannot be demonstrated by our results (Kallergis et al., 2005).

In our individuals with asthma, ∆HR and HR_peak_ during the baseline 6MWT were the only significant differences in physiological characteristics between individuals with slow or normal HRR; this simple and cheap parameter should be always evaluated in the assessment of individuals undergoing exercise training. The HRR in our study was evaluated after a 6MWT as the clinical usefulness of HRR is not dependent on maximal exercise (Cahalin et al., 2013). Also, other studies have used the 6MWT to assess the HRR in individuals with chronic respiratory diseases, confirming the usefulness of this field test (Morshedi-Meibodi et al., 2002; Lacasse et al., 2005; Swigris et al., 2009; Cholidou et al., 2014). Using the cardio-pulmonary exercise test (CPET), (Cherneva et al., 2022) have reported a higher prevalence of abnormal HRR than ours (76.5%) in individuals with mild COPD and exertional dyspnea, independent of the severity of airway obstruction. Whether this difference might be ascribed to the different exercise tests used (6MWT vs. CPET) should be evaluated by dedicated studies.

We have used a value <12 bpm as a criterion to identify slow HRR. Previous studies used values of ≤10 ≤ 12, ≤13, ≤14, or ≤16 bpm in individuals with cardiovascular and pulmonary disease (Cole et al., 1999; Zhao et al., 2021; Swigris et al., 2009; Minai et al., 2015), but a criterion to define a suitable HRR cut-off after 6MWT in COPD is not well established.

Finally, this is also the first study to show that the Bi-D (Vitacca et al., 2016; Vitacca et al., 2020) is sensitive to pulmonary rehabilitation in individuals with asthma. Future studies will evaluate the MCID of Bi-D in these individuals.

### Limitations

This is a retrospective study with the flaws of this type of study, like missing data. However, the proportion of missing data for outcome measures (MRC, Bid, CAT, and 5STS) was similar in individuals with asthma and COPD, so we are confident that this flaw has not influenced the results. On the other hand, retrospective studies on large sample sizes can give information on real-life conditions. As an example, although the multidisciplinary program included educational sessions on correct use of inhaled therapy and nurses checked the correct use, we have no data on possible misuse, which might have influenced the results.

The reported comorbidity occurrence in our study must not be considered as a real prevalence as our participants did not undergo specific diagnostic tests. The MCID of most outcome measures in individuals with asthma is not known; therefore, when lacking, we applied the MCID reported for individuals with COPD. Our data reflect a specific population of individuals undergoing pulmonary rehabilitation, and these results cannot be generalized.

## Conclusion

With the above limitations, this is the first study to evaluate the effects of pulmonary rehabilitation on HRR in individuals with asthma as compared to those with COPD. In these individuals, a pulmonary rehabilitation program, including exercise training, did not significantly improve slow HRR. The benefits of pulmonary rehabilitation were independent of HRR. No significant difference was observed between the diagnoses. Further randomized controlled studies should confirm the results of our study.

## Data Availability

The raw data supporting the conclusion of this article will be made available by the authors without undue reservation.
